# High-flow nasal oxygen vs. conventional oxygen therapy in patients with COVID-19 related acute hypoxemic respiratory failure and a do not intubate order: a multicentre cohort study

**DOI:** 10.1186/s12931-025-03231-8

**Published:** 2025-04-25

**Authors:** Daphne J. T. Sjauw, Lisa M. Hessels, Marieke L. Duiverman, Judith Elshof, Matthijs L. Janssen, Yasemin Türk, Leo Heunks, Sara J. Baart, Evert-Jan Wils, Dutch HFNO study group, Evert-Jan Wils, Yaar Aga, Hendrik Endeman, Wessel Hanselaar, Matthijs L. Janssen, Yasemin Türk, Rogier A. S. Hoek, Roxanne Heller, Dirk P. Boer, Jan H. Elderman, Alain Dubois, Oscar Hoiting, Jürgen Hölters, Marriëlle vd Steen-Dieperink, NORMO2 project group, Leo Heunks, Evert-Jan Wils, Sara J. Baart, Marieke L. Duiverman, Lucas M. Fleuren, Louise C. Urlings-Strop, Joost G. van den Aardweg, Dolf Weller, Carmen A. T. Reep, Daphne J. T. Sjauw

**Affiliations:** 1 Department of Intensive Care, Franciscus Gasthuis & Vlietland, Kleiweg 500, 3045 PM Rotterdam, The Netherlands; 2 Department of Intensive Care, Erasmus Medical Center, Rotterdam, The Netherlands; 3 Department of Respiratory Medicine, Noordwest Ziekenhuisgroep, Alkmaar, The Netherlands; 4 Department of Infectious Disease Epidemiology, Utrecht Medical Center, Utrecht, The Netherlands; 5 Department of Pulmonary Diseases / Home Mechanical Ventilation, University of Groningen, University Medical Center Groningen, Groningen, The Netherlands; 6 Groningen Research Institute for Asthma and COPD (GRIAC), University of Groningen, University Medical Center Groningen, Groningen, The Netherlands; 7 Department of Respiratory Medicine, Erasmus Medical Center, Rotterdam, The Netherlands; 8 Department of Respiratory Medicine, Franciscus Gasthuis & Vlietland, Rotterdam, The Netherlands; 9 Department of Intensive Care, Radboud University Medical Center, Nijmegen, The Netherlands; 10 Department of Biostatistics, Erasmus Medical Center, Rotterdam, The Netherlands

**Keywords:** Acute hypoxemic respiratory failure, Conventional oxygen therapy, COVID-19, Do not intubate order, High-flow nasal oxygen

## Abstract

**Background:**

High-flow nasal oxygen (HFNO) is frequently used to treat patients with acute hypoxemic respiratory failure (AHRF) due to viral pneumonia, including COVID-19. However, its clinical effect compared to conventional oxygen therapy (COT) remains largely unexplored in patients with a do not intubate (DNI) order. We aimed to assess whether HFNO compared to COT is associated with improved clinical outcomes in hospitalized patients with AHRF due to COVID-19 and a DNI order.

**Methods:**

This analysis included patients with a DNI order and SARS-CoV-2 infection, selected from three observational studies, who were treated with COT only or HFNO. The primary endpoint was in-hospital mortality, the secondary endpoint was hospital length of stay (LOS). The effect of HFNO vs. COT was assessed using multivariable regression, accounting for pre-selected confounders.

**Results:**

Between March 2020 and September 2021, 116 patients received HFNO and 110 patients received COT. Median age was 78 [72–83], and 78% of the patients had a Clinical Frailty Scale score of 4 to 9. In-hospital mortality was 64% for HFNO and 71% for COT (*p* = 0.29), with an adjusted odds ratio of 0.72 (95% confidence interval [0.34–1.54], *p* = 0.40). Hospital LOS was 11 [6–18] days for HFNO, and 7 [4–12] days for COT (*p* < 0.001), with a remaining difference after adjusting for confounders (*p* < 0.01).

**Conclusion:**

The lack of survival benefit and increased hospital LOS should be taken into account when considering HFNO for patients with a DNI order, suffering from AHRF due to viral pneumonia, like COVID-19.

***Clinical trial registration*:**

HFNO-COVID-19 study: DTR, NL9067 (Dutch Trial Registry), registration date: 27-11-2020.

**Supplementary Information:**

The online version contains supplementary material available at 10.1186/s12931-025-03231-8.

## Introduction

High-flow nasal oxygen (HFNO) is frequently used as ceiling of oxygen treatment for patients with a do not intubate (DNI) order who are suffering from acute hypoxemic respiratory failure (AHRF) due to viral pneumonia, such as caused by SARS-CoV-2 [1–6]. HFNO reduces the risk of endotracheal intubation compared to conventional oxygen therapy (COT), but does not affect mortality rates in patients with AHRF without DNI orders [7–11]. Patients with a DNI order are generally older, frailer, have more comorbidities and are at higher risk of mortality [12]. Evidence on the role of HFNO in these patients is limited to small retrospective studies lacking a comparator group using COT [1, 3, 5, 6, 13, 14]. Given their vulnerability and poor prognosis, identifying patients who may benefit from HFNO can guide clinicians in considering alternative treatment approaches, such as end-of-life or palliative care [15, 16].

This analysis aims to assess whether HFNO treatment compared to COT is associated with improved clinical outcomes in hospitalized patients with AHRF due to COVID-19 and a DNI order. Furthermore, predictors of in-hospital mortality in the HFNO group were determined using available data both prior to and during HFNO treatment.

## Methods

### Study design

This analysis selected patients with a DNI order derived from three prospective cohort studies and followed the “Strengthening the Reporting of Observational Studies in Epidemiology” (STROBE) guidelines [17].

The HFNO-COVID-19 cohort enrolled patients from 10 hospitals who received HFNO treatment during their admission between December 2020 and July 2021 [18]. This study was approved by the local Medical Ethics Committee (MEC-U number W20.283) and registered in the Dutch Trial Registry (DTR, NL9067). The Northwest Hospital group (NWZ) cohort was a single-centre study conducted between March 2020 and September 2021, including patients receiving either HFNO treatment or COT only [19]. This study was approved by the institutional research committee of the Northwest clinics (number L020 – 115). The University Medical Center Groningen (UMCG) cohort was a single-centre study and included patients receiving HFNO treatment or COT only between March 2020 and October 2021 [20]. The study was approved by the local Medical Ethics Committee (METc number 2020/573). All studies have been carried out in accordance with the Declaration of Helsinki. Written informed consent was waived due to the observational nature of the studies. However, some participating hospitals obtained written informed consent if required by local guidelines of institutional research committees.

### Study population and setting

Patients were eligible for inclusion if they met the following criteria: (1) age ≥ 18 years, (2) positive SARS-CoV-2 test, (3) DNI order (no endotracheal intubation) at any time point during hospital admission decided at the discretion of the treating medical team and/or based on patient’s preferences, and (4) treatment with either COT only or HFNO as ceiling of oxygen treatment with a minimum flow rate of 6 L oxygen per minute (L/min). Exclusion criteria were: (1) endotracheal intubation, (2) transfer from/to non-participating facilities and/or (3) inability to provide informed consent.

Details of local practices during the study period are depicted in Supplementary Table 1. All patients started COT and/or HFNO on the ward, except for 5 centres where HFNO was not available on the ward. The nurse-to-patient ratio on the ward varied between centres from 1:3 to 1:4 during day-shifts and from 1:5 to 1:10 during night-shifts. Vital signs monitoring was more frequent in the first 24 h of therapy and 3 to 6 times daily thereafter. The HFNO flow rate at initiation was 35–60 L/min, maximum flows were 40–60 L/min, HFNO was applied continuously and HFNO phasing out started at a flow rate between 30 and 40 L/min.

### Outcomes, data collection, and preparation

The primary outcome was in-hospital mortality, the secondary outcome was hospital length of stay (LOS) in days, and the tertiary outcome was the cause of death. The outcome of prediction was in-hospital mortality in patients treated with HFNO.

Data was prospectively collected [18, 19] and supplemented with data collected retrospectively specific for the current analysis using predefined similar definitions (Supplementary Table 2). The Clinical Frailty Scale (CFS) score was categorized as fit (CFS 1–3), vulnerable (CFS 4–5) and frail (CFS 6–9) [21]. Respiratory parameters (respiratory rate, oxygen saturation (SpO_2_) and details regarding the oxygen therapy modality and flow) were recorded at multiple time points: just prior to and after HFNO initiation (0, 0.5, 1, 2, 6, 12 and 24 h). Fraction of inspired oxygen (FiO_2_) on COT was estimated using the equation: FiO_2_ = 21% + oxygen flow rate in L/min × 3 [22], and further categorized into: group 1) room air, group 2) nasal oxygen 1–6 L/min or air-entrainment mask 10 L/min, group 3) air-entrainment mask 15 L/min or non-rebreathing mask 10 L/min and group 4) non-rebreathing mask 15 L/min (Supplementary Table 3).

### Statistical analyses

A convenient sample approach was used. Categorical variables were reported as numbers (percentages) and continuous variables as medians [25th–75th percentile]. Differences between patients on HFNO vs. COT, and between survivors and non-survivors among HFNO patients, were analysed using the Mann–Whitney U-test for continuous variables and the Chi-squared test for categorical variables. A *p*-value of <0.05 was considered statistically significant. If <5% of the variables were missing, complete case analyses were performed. Otherwise, missing data were imputed 10 times, and results were pooled following the Rubin’s rules [23].

#### HFNO versus COT

To assess the difference of in-hospital mortality between HFNO and COT, unadjusted pairwise comparisons and multivariable logistic regression analyses were used, with odds ratios (OR) presented alongside 95% confidence intervals (CI). Relevant confounders for the multivariable logistic regression analyses were pre-selected based on literature review and clinical expertise (Supplementary Table 4). The number of confounders was limited by the rule of thumb of 10 events per variable. Selected confounders were age, CFS score, dexamethasone treatment, and respiratory parameters prior to (potential) HFNO initiation (respiratory rate, SpO_2_, and FiO_2_). Because of variability in HFNO initiation thresholds [1, 3–6, 13, 14, 24, 25] and its potential impact on outcomes, two approaches were used to select respiratory confounders closest to when patients receiving COT would have initiated HFNO treatment. In the “early” approach, variables were selected when patients required ≥6 L/min of COT, and in the “maximum” approach, they were selected at the maximum level of COT.

Time from the (potential selected) initiation of HFNO until in-hospital mortality up to 28 days was evaluated using the Kaplan–Meier method, and tested with the log-rank test.

Hospital LOS was logarithmically transformed because of its non-normal distribution. Multivariable linear regression analysis was used, adjusting for previous selected confounders focusing on hospital admission. Estimates are presented with 95% CI.

#### Potential predictors for in-hospital mortality in the HFNO group

To determine predictors of in-hospital mortality in patients treated with HFNO, measurements at two relevant timeframes were selected: prior to HFNO initiation and after HFNO initiation.

For the prediction using measurements prior to HFNO initiation, we first assessed the differences through unadjusted pairwise comparisons of baseline characteristics and respiratory parameters. Secondly, we assessed the independent association of predefined variables (respiratory rate, SpO_2_, FiO_2_, all measured prior to HFNO initiation) using multivariable logistic regression analyses. The discriminative performance of this multivariable model was evaluated using the Area Under the Receiver Operating Characteristic curve (AUROC), presented with 95% CI. AUC’s from univariable analyses for the SpO_2_/FiO_2_ (S/F) ratio and ROX index (S/F ratio divided by respiratory rate)[26] were added.

To determine predictors measured during HFNO treatment, similar analyses strategies were performed using measurements at different time points within the first 24 h after HFNO initiation. The course of respiratory rate, oxygen saturation, S/F ratio, and ROX index over time between survivors and non-survivors were displayed via boxplots.

All analyses were performed using R (version 4.2.1). We used R package “survival” to plot the Kaplan–Meier curve, “MICE” for multiple imputation and pooling the imputed datasets afterwards, “psfmi” for the estimation of the multivariable models to predict in-hospital mortality and “pROC” to evaluate discriminative performances of the models.

## Results

### HFNO versus COT

**Fig. 1:**
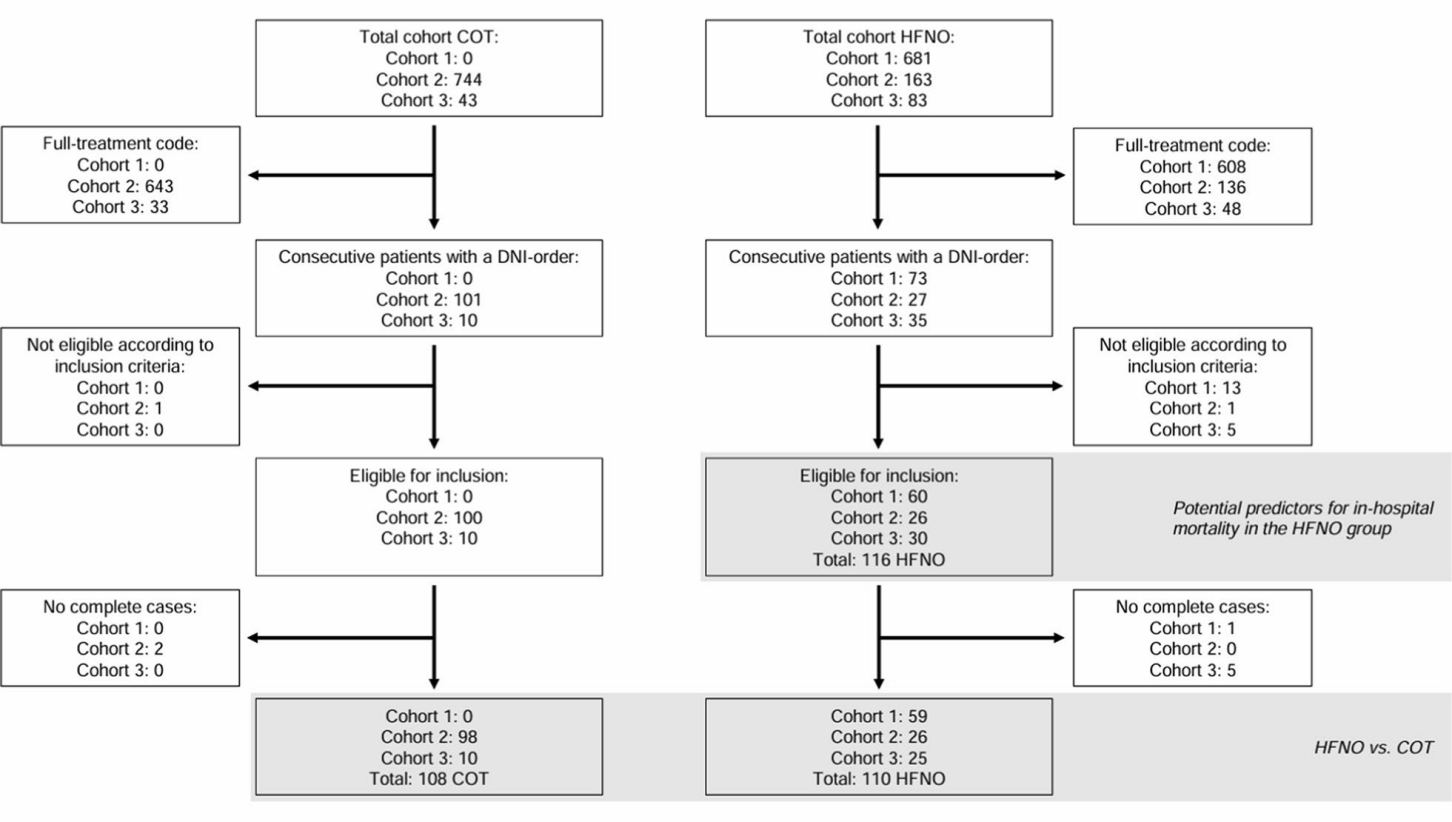
Flowchart study population. Cohort 1 refers to the HFNO-COVID-19 cohort, cohort 2 refers to the Northwest Hospital group (NWZ) cohort, cohort 3 refers to the University Medical Center Groningen (UMCG) cohort. *HFNO* high-flow nasal oxygen, *COT* conventional oxygen therapy

To assess the effect of HFNO compared to COT on in-hospital mortality, 218 out of 226 available patients (HFNO n = 110, COT n = 108) were selected (Fig. 1).

Patient characteristics are presented in Table 1 (and Supplementary Table 5). Median age was 78 [72–83], 62% were male, and 78% had a CFS score between 4 and 9. Patients on HFNO were younger, less frail, and more frequently treated with dexamethasone.

**Table 1:** Characteristics of the total study cohort, and divided by HFNO and COT

	All patients (n = 218)	HFNO (n = 110)	COT (n = 108)	*p*-value*
Demographics at hospital admission				
Age (years)	78 [72–83]	75 [69–80]	80 [75–85]	<0.001
Sex (male, n (%))	135 (62)	69 (63)	66 (61)	0.92
Body Mass Index (kg/m^2^)	27 [24–31]	28 [24–32]	26 [24–29]	0.15
Charlson Comorbidity Index (n (%))				0.11
0	19 (9)	14 (13)	5 (5)	
1	75 (34)	36 (33)	39 (36)	
≥2	124 (57)	60 (55)	64 (59)	
Clinical Frailty Scale (n (%))				<0.001
Fit (1–3)	47 (22)	37 (34)	10 (9)	
Vulnerable (4–5)	101 (46)	41 (37)	60 (56)	
Frail (6–9)	70 (32)	32 (29)	38 (35)	
Laboratory at hospital admission				
CRP (mg/mL)	94 [48–148]	90 [49–141]	95 [46–154]	0.73
Urea (mmol/L)	9.2 [6.6–12.3]	8.6 [6.2–11.6]	9.7 [7.5–12.8]	0.02
Platelet count (10^9^/L)	175 [129–231]	177 [130–232]	169 [131–228]	0.98
Lymphocyte count (10^9^/L)	0.70 [0.54–0.90]	0.70 [0.50–0.90]	0.80 [0.60–1.00]	0.02
Treatment during hospital admission				
Dexamethasone (n (%))	190 (87)	106 (96)	84 (78)	<0.001
Interleukin-6 receptor blockers (n (%))	30 (14)	23 (21)	7 (7)	0.004
Prior to start HFNO or COT at ≥ 6 L/min				
Hours from hospital admission	46.1 [3.8–103.1]	53.3 [7.4–126.2]	35.3 [1.2–90.8]	0.02
Respiratory rate (breaths/minute)	28 [22–32]	28 [23–32]	28 [22–32]	0.55
SpO_2_ (%)	93 [90–95]	92 [89–94]	94 [93–95]	<0.001
FiO_2_	0.60 [0.40–0.66]	0.66 [0.60–0.66]	0.40 [0.39–0.66]	<0.001
FiO_2_ categories (n (%))^‡^				<0.001
2	84 (39)	18 (16)	66 (61)	
3	34 (16)	20 (18)	14 (13)	
4	100 (46)	72 (66)	28 (26)	
S/F ratio	152 [141–233]	144 [136–155]	231 [150–238]	<0.001
ROX index	6.4 [4.8–8.6]	5.4 [4.4–6.9]	7.7 [5.4–10.0]	<0.001
Prior to start HFNO or COT at maximal level				
Hours from hospital admission	60.3 [15.4–124.5]	53.3 [7.4–126.2]	64.7 [23.8–122.3]	0.20
Respiratory rate (breaths/minute)	28 [22–32]	28 [23–32]	26 [22–32]	0.42
SpO_2_ (%)	94 [90–96]	92 [89–94]	95 [93–97]	<0.001
FiO_2_	0.66 [0.60–0.66]	0.66 [0.60–0.66]	0.66 [0.60–0.66]	0.13
FiO_2_ categories (n (%))^‡^				0.13
2	26 (12)	18 (16)	8 (7)	
3	42 (19)	20 (18)	22 (20)	
4	150 (69)	72 (66)	78 (73)	
S/F ratio	145 [139–154]	144 [136–155]	145 [142–152]	0.13
ROX index	5.5 [4.6–7.0]	5.4 [4.4–6.9]	5.6 [4.7–7.1]	0.50
Outcomes				
In-hospital mortality (n (%))	147 (67)	70 (64)	77 (71)	0.29
Hospital length of stay [median, IQR]				
All	9 [5–16]	11 [6–18]	7 [4–12]	0.001
Survivors	15 [10–21	18 [11–23]	13 [8–18]	
Non-survivors	6 [4–11]	8 [4–14]	6 [3–10]	

Data presented as median [interquartile ranges], unless denoted otherwise

*Using Mann–Whitney U-test for continuous variables and Chi-square test for categorical variables

^‡^FiO_2_ divided into three categories: group 0) room air, group 1) nasal oxygen 1–6 L/min or air-entrainment mask 10 L/min, group 2) air-entrainment mask 15 L/min or non-rebreathing mask 10 L/min and group 3) non-rebreathing mask 15 L/min

*COT* conventional oxygen therapy, *CRP* C-reactive protein, *FiO*_2_ fraction of inspired oxygen, *HFNO* high-flow nasal oxygen, *IQR* interquartile range, *L* litres, *min* minute, *N* number, *ROX index* respiratory oxygenation index (SpO_2_/FiO_2_/respiratory rate), *SpO*_2_ oxygen saturation, *S/F ratio* SpO_2_/FiO_2_ ratio

In-hospital mortality rate was 67%, predominantly attributed to progressive respiratory failure (96%). All patients died within 28 days after admission, except one patient in the HFNO group (day 37). Unadjusted in-hospital mortality rates were 64% in the HFNO group and 71% in the COT group (*p* = 0.29; Table 1).

In the HFNO group, the respiratory rate prior to HFNO initiation was 28 [23–32], SpO_2_ level was 92 [89–94], and the FiO_2_ level was high (66% on 15 L/min non-rebreathing mask, Table 1).

**Fig. 2:**
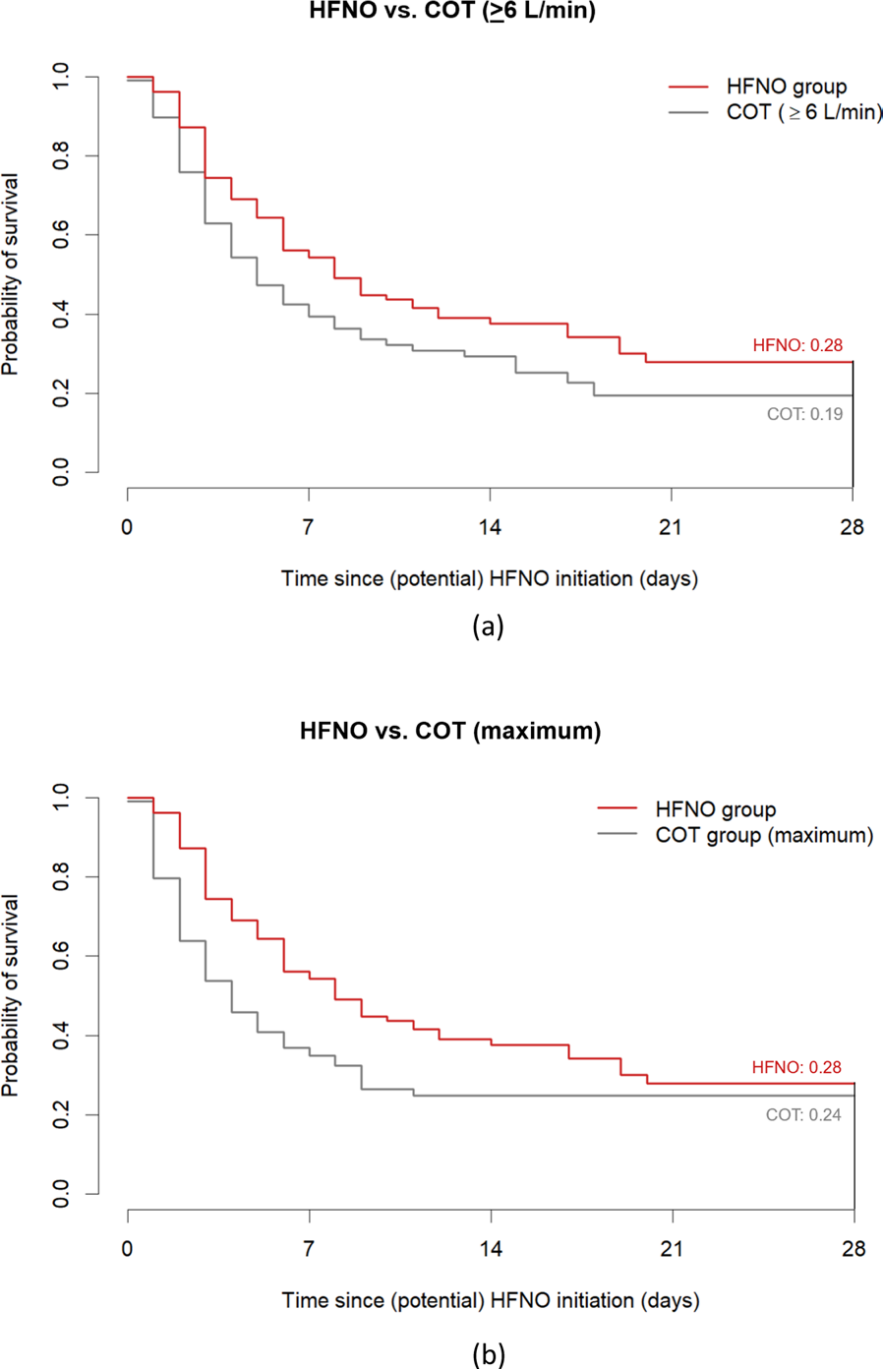
Kaplan–Meier curve of HFNO vs. COT. *HFNO* high-flow nasal oxygen, *COT* conventional oxygen therapy. **a** Kaplan–Meier Curve of HFNO vs. COT (time point selected as potential HFNO initiation point for COT: at least 6 L oxygen per minute), log-rank *p*-value: 0.02. **b** Kaplan–Meier Curve of HFNO vs. COT (time point selected as potential HFNO initiation point for COT: at maximum oxygen therapy level during hospital admission). Log-rank *p*-value: 0.002

For the COT group, we considered “early” and “maximum” approaches to select the time point when patients would have met the potential HFNO treatment eligibility criteria. In the early approach, respiratory rates were similar to patients receiving HFNO (28 [22–32]). SpO_2_ levels were higher (94 [93–95]) and FiO_2_ levels were lower. In the multivariable analysis, HFNO compared to COT was not associated with in-hospital mortality (OR 0.72 [0.34–1.54]; Table 2). The survival time was longer for the HFNO group (log-rank *p*-value = 0.02; Fig. 2a).

**Table 2:** Odds ratio’s for in-hospital mortality at time points COT ≥ 6 L/min or at maximum level

Prior to (potential) HFNO initiation	COT at ≥6 L/min		COT at maximal level	
	OR [95% CI]	*p*-value	OR [95% CI]	*p*-value
HFNO vs. COT	0.72 (0.34–1.54)	0.40	0.85 (0.40–1.78)	0.66
*Confounders*				
Age (per 1-year increase)	1.03 (0.99–1.07)	0.20	1.03 (0.99–1.07)	0.21
Clinical frailty scale*				
Vulnerable vs. Fit	2.06 (0.93–4.56)	0.08	2.46 (1.06–5.72)	0.04
Frail vs. Fit	1.20 (0.54–2.68)	0.66	1.57 (0.67–3.71)	0.30
Dexamethasone (yes/no)	0.58 (0.21–1.60)	0.29	0.69 (0.24–1.99)	0.50
SpO_2_ (per 1%-point increase)	0.90 (0.83–0.98)	0.02	0.90 (0.82–0.98)	0.02
FiO_2_^‡^				
Category 3 vs. 2	0.81 (0.33–2.01)	0.65	1.35 (0.47–3.85)	0.57
Category 4 vs. 2	1.43 (0.68–3.03)	0.35	4.75 (1.86–12.13)	<0.01
RR (per 1-breath/min increase)	1.01 (0.96–1.05)	0.76	1.02 (0.97–1.07)	0.43

Multivariable logistic regression analysis for outcome in-hospital mortality

*Clinical Frailty Scale was divided in category 1) fit 1–3, category 2) vulnerable 4–5, category 3) frail 6–9. ^‡^FiO_2_: estimated fraction of inspired oxygen, divided into three categories: group 1) room air, group 2) nasal oxygen 1–6 L/min or air-entrainment mask 10 L/min, group 3) air-entrainment mask 15 L/min or non-rebreathing mask 10 L/min and group 4) non-rebreathing mask 15 L/min

*CI* confidence interval, *COT* conventional oxygen therapy, *HFNO* high-flow nasal oxygen, *OR* odds ratio, *RR* respiratory rate, *SpO*_2_ oxygen saturation

In the maximum approach, respiratory rates (26 [22–32]) and FiO_2_ levels were similar to the HFNO group, and SpO_2_ levels were higher (95 [93–97]). In the multivariable analysis, HFNO was not associated with in-hospital mortality (OR 0.85 [0.40–1.78]; Table 2). The survival time was longer for the HFNO group (log-rank *p*-value = 0.002; Fig. 2b).

Hospital LOS was longer in the HFNO group compared to the COT group (11 [6–18] vs. 7 [4–12], *p* = 0.001), observed in both survivors and non-survivors (Table 1). The estimate for hospital LOS adjusted for confounders was 0.34 ([0.13–0.55]; Supplementary Table 6).

### Potential predictors for in-hospital mortality in the HFNO group

To determine predictors of in-hospital mortality in patients treated with HFNO, a total of 116 patients were selected (Fig. 1). We focused on two relevant time frames for the measurements of predictors: prior to HFNO initiation and during HFNO treatment.

For the measurements taken prior to HFNO initiation, unadjusted pairwise comparisons showed no differences in characteristics between survivors and non-survivors (Supplementary Table 7). Multivariable logistic regression analysis identified SpO_2_ level as a predictor of in-hospital mortality (multivariable model: AUC 0.71 [0.60–0.80]). Univariable AUC’s for the S/F ratio and ROX index were 0.70 [0.59–0.79] and 0.63 [0.52–0.73], respectively (Supplementary Table 8).

**Fig. 3:**
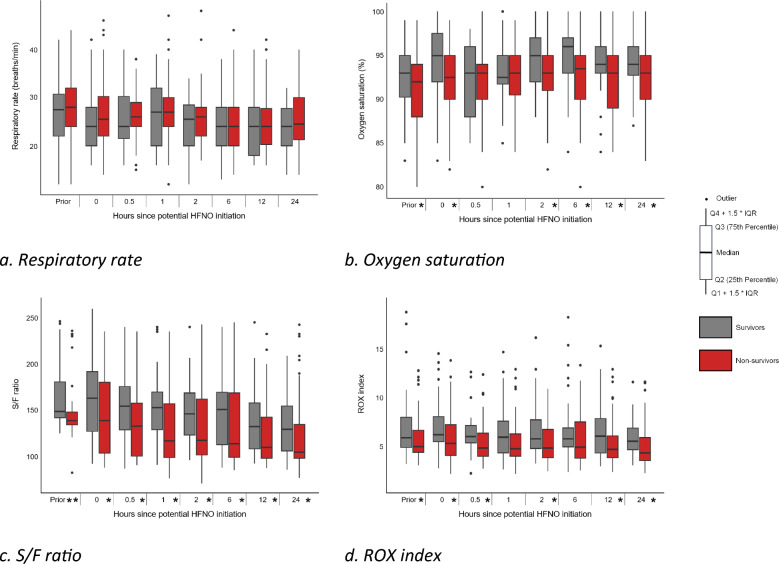
Course of respiratory parameters between survivors and non-survivors. **a** Respiratory rate, **b** Oxygen saturation, **c** S/F ratio, **d** ROX index. Parameters are depicted as Boxplot, *p*-values: Mann–Whitney U-test for continuous variables and Chi-square test for categorical variables. * represent *p*-values < 0.05, ** represent *p*-values < 0.001. *T* time point, *prior* prior to HFNO initiation, *Q* quartile, *IQR* interquartile range, *S/F ratio* SpO_2_/FiO_2_ ratio, *ROX index* respiratory oxygenation index (SpO_2_/FiO_2_/respiratory rate)

The course of respiratory parameters within the first 24 h after HFNO initiation stratified by survival status is depicted in Fig. 3. Significant differences in unadjusted pairwise analyses between survivors and non-survivors were observed for SpO_2_ levels at several time points (prior to, 0, 2, 6, 12, and 24 h), but the absolute differences were small (median differences ranged 1–2%). S/F ratio (median differences range 10–37) and ROX index (median differences range 0.9–1.3) differed at most time points. Multivariable logistic regression analyses identified SpO_2_ level as a predictor for in-hospital mortality at several time points (0, 2, and 6 h), and the level of FiO_2_ was associated at 24 h. AUC’s based on univariable models for the S/F ratio and ROX index ranged from 0.61 to 0.70, and 0.58 to 0.68 respectively (Supplementary Table 8).

## Discussion

Evidence supporting the use of HFNO in patients with DNI orders is limited and often of low quality [27]. In this analysis using data from three observational cohort studies, we assessed the role of HFNO in patients with a DNI order suffering from AHRF due to COVID-19. Our main findings can be summarized as follows: First, in-hospital mortality was high (67%) and primarily due to respiratory failure. Second, HFNO was not associated with a survival benefit compared to COT but was associated with a prolonged hospital stay. Finally, prediction accuracy for in-hospital mortality at HFNO initiation or during HFNO treatment was moderate.

Overall, in-hospital mortality rate in this population was high (67%) which aligns with mortality rates in similar study populations (ranging between 58 and 86%) [1, 3–6, 13, 14]. In adjusted analyses, correcting for differences in patient characteristics, we observed no associated survival benefit of HFNO over COT. This finding was consistent for the two HFNO eligibility time points in the COT group. HFNO has previously been suggested as a treatment for this population based on retrospective observational studies [1–6, 13]. These studies were mostly descriptive, predominantly lacked a COT comparator group, and consisted of small sample sizes. Only one study compared HFNO with COT using propensity matching, and reported a 30-day survival rate of 17 vs. 8%, respectively [14]. Its retrospective, single-centre design, small sample size (n = 67), inclusion of only patients > 75 years, the use of propensity score matching in only a small sample [28] and lack of sufficient methodological details limit the study’s robustness and generalizability. In our study, data were more granular and prospectively collected, and the sample size was relatively large.

We further observed a prolonged hospital LOS in both surviving and non-surviving patients on HFNO. The consequence of a lack of a clear survival benefit at hospital discharge, combined with prolonged hospital LOS, should be considered in the context of societal and individual costs and benefits. Nevertheless, HFNO may still play a role in alleviating symptoms like dyspnoea and discomfort, although evidence remains uncertain [14, 25, 29, 30]. From a palliative care perspective, reducing such symptoms and creating time for end-of-life care can be important goals. Our study provides insights that can help balance these treatment goals, which may be especially relevant for resource-intensive strategies during times of resource scarcity, such as future pandemics.

Guiding such clinical decision-making can be facilitated by predicting who may benefit from HFNO [15, 16]. In our study, baseline characteristics, respiratory variables or indicators of disease severity were not different between survivors and non-survivors. In contrast, previous studies found differences in characteristics such as age [5], sex [6], CFS score or WHO performance status at admission [4], and (the number of) comorbidities [1, 5, 6], based on unadjusted comparisons. In our multivariable logistic regression analysis, only SpO_2_ level appeared to be a predictor of in-hospital mortality, but absolute differences were small. Composite indices, such as the S/F ratio and ROX index, have fair discriminative accuracy to predict intubation in full-code patients on HFNO [26, 31, 32]. However, their performance in DNI patients is understudied [4]. In our analyses, the accuracy of these indices was moderate at best. Collectively, these findings suggest that other variables may be more relevant in this specific population. Variables capturing the patient’s premorbid status, such as the Charlson Comorbidity Index [33] and CFS score [34], or inflammatory variables are obvious candidates, but these variables were not associated with in-hospital mortality in the current analyses.

Our study has several strengths. We included one of the largest cohorts of the understudied patients with a DNI order and AHRF due to COVID-19, assessing the impact of HFNO on both in-hospital mortality and hospital LOS. Furthermore, this study surpassed the descriptive nature of previous studies [1, 3–6, 13], using both unadjusted and adjusted analyses.

There are also limitations to consider. First, this analysis was based on observational data and can still be hampered by residual biases, despite the efforts to include most relevant confounders for the outcome of interest. Some supplemented data was collected retrospectively, which could have led to information bias. However, most of the data was collected prospectively and standardized data collection definitions were used. To substantiate the conclusions and further limit biases, a randomized controlled trial is required. Second, the sample size was insufficient to include a wider range of variables for prediction modelling and may have caused imprecision to detect small treatment differences between HFNO and COT. Future studies should strive for larger sample sizes to explore other relevant variables in this specific population. Third, the three cohorts originated from overlapping but slightly different time periods. As the administration of steroids was one of the major improvements in COVID-19 treatment [35], by including steroid use as confounder the analysis was at least partly corrected for differences in treatment strategies. Fourth, although mortality and length of stay are relevant clinical outcomes for studies on HFNO, more palliative endpoints such as comfort and dyspnoea are arguably equally relevant in this specific frail population. Finally, in this study patients with COVID-19 were exclusively included, which may limit generalizability to other forms of acute hypoxemic respiratory diseases. Nonetheless, the current data may encourage further exploration of the role of HFNO in other hypoxemic diseases and consider patients with a DNI order as a relevant pre-defined subgroup.

## Conclusions

HFNO in patients with a DNI order and AHRF due to viral pneumonia (COVID-19) was not associated with a survival benefit compared to COT, but with prolonged hospital LOS. Decisions on initiating HFNO treatment in these patients should consider its potential impact on in-hospital mortality and hospital LOS, as well as patient-centred outcomes such as relief from dyspnoea, alleviation of discomfort, and palliative care needs. Such considerations are especially relevant in resources-limited settings but also apply to individual patient care in general. Future studies on the role of HFNO in other hypoxemic diseases should specifically focus on frail patients with DNI orders and include not only outcomes as mortality, but also comfort and dyspnoea.

## Supplementary Information

Additional file 1.

## Data Availability

No datasets were generated or analysed during the current study.

## Abbreviations

AHRF: Acute hypoxemic respiratory failure
AUC: Area under the curve
BMI: Body mass index
CFS: Clinical frailty scale
CI: Confidence interval
COT: Conventional oxygen therapy
COVID-19: Coronavirus disease 2019
DNI: Do not intubate
FiO_2_: Fraction of inspired oxygen
HFNO: High-flow nasal oxygen
L/min: Litres per minute
LOS: Length of stay
OR: Odds ratio
*p*: *p*-Value
ROX index: Respiratory oxygenation index
RR: Respiratory rate
SpO_2_: Oxygen saturation via pulse oximetry
