# Supplementary material for: High-flow nasal oxygen vs. conventional oxygen therapy in patients with COVID-19 related acute hypoxemic respiratory failure and a do not intubate order: a multicentre cohort study
Source: Respir Res. 2025 Apr 25;26:161. doi: 10.1186/s12931-025-03231-8 (PMC12032817; doi:10.1186/s12931-025-03231-8)
Supplement: Supplementary file 1 — Additional file 1. [file 12931_2025_3231_MOESM1_ESM.docx]

**Supplementary**

# **Table 1. Local practices in participating centres during study period**

|  | NWZ | UMCG | HFNO-COVID-19* |
| --- | --- | --- | --- |
| Bed capacity (beds with HFNO) | 32 (10) | 14 (10) | 12 to 36 (2 to 12) |
| Vital parameter control | COT: every 6h  HFNO: first 6h every 2h, afterwards every 6h | COT and HFNO: first 30 min 2 times, first 2h 4 times, afterwards every 3h | HFNO: first 24h at 0.5, 1, 2, 4, 6, 12 and 24h, then 3 to 6 times daily |
| Continuous monitoring availability | No | Only for SpO_2_ | SpO_2_ and RR in 4 out of 7 wards |
| Indication IL-6 RB | If O_2_ flow >6 L/min, and/or if CRP ≥75 mg/ml | If O_2_ flow >6 L/min, and/or if CRP ≥75 mg/ml | If oxygen ≥6 L/min, and/or if CRP ≥75 mg/ml |
|  |  |  |  |
| *Personnel availability (day-night shift)* |  |  |  |
| Nurse:patient ratio | 1:4-1:10 | 1:3-1:5 | 1:3 to 1:4 - 1:5 to 1:7 |
| Consultants | 1-1 | 1-1 | 1 to 2 - 1 to 2 |
| Residents | 3-1 | 2-1 | 1 to 3 - 1 to 2 |
|  |  |  |  |
| *HFNO* |  |  |  |
| Criteria to initiate HFNO | Not able to reach SpO_2_ >94% with 10 L/min | SpO_2_ <92% with 60% air-entrainment mask | SpO_2_ <92% and/or RR >30/min despite treatment with at least 6 L/min O_2_ |
| Device shortage | No | No | No |
| Type of HFNO cannula interface | Symmetrical | Symmetrical | Symmetrical |
| Flow rate start | 35 L/min | 40 L/min | 40-60 L/min |
| Flow rate limit | 60 L/min | 60 L/min | 40-60 L/min |
| HFNO usage (intermittent/ continuous) | Continuous | Continuous | Continuous |
| Flow rate criterion for phasing out HFNO | 30 L/min | 40 L/min | 30-40 L/min |
| *Ranges are given because the HFNO-COVID-19 cohort included 10 centres. NWZ: Northwest Hospital group cohort, UMCG: University Medical Center Groningen cohort. Abbreviations: COT: conventional oxygen therapy, CRP: C-reactive protein, DNI: do not intubate order, h: hour(s), HFNO: high-flow nasal oxygen, IL-6RB: interleukine-6 receptor blockers, L: litres, min: minute, O_2_: oxygen, RR: respiratory rate, SpO_2_: oxygen saturation. | | | |

# **Table 2. Data collection and definitions**

| Variable | Definition | Measurement scale | Prospectively/retrospectively collected |
| --- | --- | --- | --- |
| *Collected at hospital admission* |  |  |  |
| Age | In years | Continuous | Cohort 1: prospectively  Cohort 2: prospectively  Cohort 3: prospectively |
| Sex | Male, female | Binary | Cohort 1: prospectively  Cohort 2: prospectively  Cohort 3: prospectively |
| Body Mass Index (BMI) | In kilograms/meter^2^ | Continuous | Cohort 1: prospectively  Cohort 2: prospectively  Cohort 3: prospectively |
| Days ill since symptom onset | Estimated days since first subjective symptoms | Continuous | Cohort 1: prospectively  Cohort 2: prospectively  Cohort 3: prospectively |
| Number of comorbidities (0, 1, or 2 and more) | According to Charlson Comorbidity Index (1) (Myocardial infarction, congestive heart failure, peripheral vascular disease, cerebral vascular accident (CVA)/transient ischemic attack (TIA), dementia, chronic obstructive pulmonary disease (COPD), connective tissue disease, peptic ulcer disease, liver disease, diabetes mellitus, hemiplegia, moderate to severe chronic kidney disease, solid tumour, leukaemia, lymphoma, AIDS) | Ordinal | Cohort 1: prospectively  Cohort 2: prospectively  Cohort 3: retrospectively |
| Clinical Frailty Scale (2) | According to 1) Very fit, 2) Well, 3) Managing well, 4) Vulnerable, 5) Mildly frail, 6) Moderately frail, 7) Severely frail, 8) Very severely frail, 9) Terminally ill | Ordinal | Cohort 1: partly retrospectively  Cohort 2: retrospectively  UMC cohort: prospectively |
| C-reactive protein (CRP) | In milligrams/millilitres | Continuous | Cohort 1: prospectively  Cohort 2: prospectively  Cohort 3: prospectively |
| Urea | In mmol/litres | Continuous | Cohort 1: prospectively  Cohort 2: prospectively  Cohort 3: retrospectively |
| Platelet count | In 10^9^/litres | Continuous | Cohort 1: prospectively  Cohort 2: prospectively  Cohort 3: retrospectively |
| Lymphocyte count | In 10^9^/litres | Continuous | Cohort 1: prospectively  Cohort 2: prospectively  Cohort 3: retrospectively |
| Glasgow Coma Scale |  | Score | Cohort 1: prospectively  Cohort 2: prospectively  Cohort 3: retrospectively |
| Respiratory rate | In breaths/minute | Continuous | Cohort 1: prospectively  Cohort 2: prospectively  Cohort 3: prospectively |
| Oxygen saturation | In percentage (%) | Continuous | Cohort 1: prospectively  Cohort 2: prospectively  Cohort 3: prospectively |
| Details regarding the modality and flow of oxygen therapy | Room air, nasal cannula 1-6 L/min, air entrainment mask 6, 10 and 15 L/min, non-rebreathing mask 10 and 15 L/min | Ordinal | HFNO-COVID-19: prospectively  Cohort 2: retrospectively  Cohort 3: retrospectively |
| *During hospital admission* |  |  |  |
| Dexamethasone treatment | Yes/no | Binary | Cohort 1: prospectively  Cohort 2: prospectively  Cohort 3: prospectively |
| Interleukin-6 receptor receptor blockers treatment | Yes/no | Binary | Cohort 1: prospectively  Cohort 2: prospectively  Cohort 3: prospectively |
| *Prior to HFNO initiation* |  |  |  |
| Respiratory rate | In breaths/minute | Continuous | HFNO-COVID-19: prospectively  Cohort 2: prospectively (for HFNO), retrospectively (for COT)  Cohort 3: prospectively (for HFNO), retrospectively (for COT) |
| Oxygen saturation | In percentage (%) | Continuous | HFNO-COVID-19: prospectively  Cohort 2: prospectively (for HFNO), retrospectively (for COT)  Cohort 3: prospectively (for HFNO), retrospectively (for COT)) |
| Details regarding the modality and flow of oxygen therapy | Room air, nasal cannula 1-6 L/min, air entrainment mask 6, 10 and 15 L/min, non-rebreathing mask 10 and 15 L/min | Ordinal | HFNO-COVID-19: prospectively  Cohort 2: retrospectively  Cohort 3: retrospectively |
| *At different time points after HFNO initiation** |  |  |  |
| Respiratory rate | In breaths/minute | Continuous | Cohort 1: prospectively  Cohort 2: retrospectively  Cohort 3: prospectively |
| Oxygen saturation | In percentage (%) | Continuous | Cohort 1: prospectively  Cohort 2: retrospectively  Cohort 3: prospectively |
| Details regarding the modality and flow of oxygen therapy | Yes/no | Ordinal | Cohort 1: prospectively  Cohort 2: retrospectively  Cohort 3: prospectively |
| *Outcomes* |  |  |  |
| In-hospital mortality | Yes/no | Binary | Cohort 1: prospectively  Cohort 2: prospectively  Cohort 3: prospectively |
| Hospital length of stay | In days | Continuous | Cohort 1: prospectively  Cohort 2: prospectively  Cohort 3: prospectively |
| Cause of death | Respiratory failure, multi-organ failure, myocardial infarction, thrombotic event (cerebral vascular accident, pulmonary event), heart arrhythmia, other | Categorical | Cohort 1: prospectively  Cohort 2: prospectively  Cohort 3: retrospectively |
| * Time points: 0 (= immediately after HFNO initiation), 0.5 hours, 1 hour, 2 hours, 6 hours, 12 hours and 24 hours. Abbreviations: COT: conventional oxygen therapy, HFNO: High-Flow Nasal Oxygen, L/min: liters per minute. | | | |

# **Table 3. Oxygen device to estimated FiO_2_ and division into FiO_2_ category**

| **Mode of oxygen therapy** | **Estimated FiO_2_** | **FiO_2_ Category** |
| --- | --- | --- |
| Room air | 0.21 | 1 |
| 1 L/min | 0.24 | 2 |
| 2 L/min | 0.27 | 2 |
| 3 L/min | 0.30 | 2 |
| 4 L/min | 0.33 | 2 |
| 5 L/min | 0.36 | 2 |
| 6 L/min | 0.39 | 2 |
| Air-entrainment mask orange 6 L/min | 0.31 | 2 |
| Air-entrainment mask red 10 L/min | 0.40 | 2 |
| Non-Rebreathing Mask 10 L/min | 0.51 | 3 |
| Air-entrainment mask green 15 L/min | 0.60 | 3 |
| Non-Rebreathing Mask 15 L/min | 0.66 | 4 |
|  |  |  |
| Estimated FiO_2_ = 21% + oxygen flow rate in litre/min x 3.  Abbreviations: FiO_2_: Fraction of inspired oxygen, HFNO: High-Flow Nasal Oxygen,  L: litres, min: minute. | | |

# **Table 4. Pre-selected confounding factors: Rationale**

|  | Literature |
| --- | --- |
| Age | - Zhou F, Yu T, Du R, Fan G, Liu Y, Liu Z, et al. Clinical course and risk factors for mortality of adult inpatients with COVID-19 in Wuhan, China: a retrospective cohort study. Lancet. 2020;395(10229):1054-62 - Biswas M, Rahaman S, Biswas TK, Haque Z, Ibrahim B. Association of Sex, Age, and Comorbidities with Mortality in COVID-19 Patients: A Systematic Review and Meta-Analysis. Intervirology. 2020:1-12. - Williamson EJ, Walker AJ, Bhaskaran K, Bacon S, Bates C, Morton CE, et al. Factors associated with COVID-19-related death using OpenSAFELY. Nature. 2020;584(7821):430-6. - Grasselli G, Zangrillo A, Zanella A, Antonelli M, Cabrini L, Castelli A, et al. Baseline Characteristics and Outcomes of 1591 Patients Infected With SARS-CoV-2 Admitted to ICUs of the Lombardy Region, Italy. JAMA. 2020;323(16):1574-81. - Van Steenkiste J, van Herwerden MC, Weller D, van den Bout CJ, Ruiter R, den Hollander JG, et al. High-flow Nasal Cannula therapy: A feasible treatment for vulnerable elderly COVID-19 patients in the wards. Heart Lung. 2021;50(5):654-9. |
| Clinical Frailty Scale | - Rockwood K, Song X, MacKnight C, Bergman H, Hogan DB, McDowell I, et al. A global clinical measure of fitness and frailty in elderly people. CMAJ. 2005;173(5):489-95. - Smesseim I, Mooij-Kalverda K, Hessels L, Korevaar DA, Atasever B, de Graaff H, et al. High flow nasal cannula for acute respiratory failure due to COVID-19 in patients with a 'do-not-intubate' order: A survival analysis. Clin Respir J. 2023;17(2):115-9. - Meersseman C, Grolleau E, Freymond N, Wallet F, Gilbert T, Locatelli-Sanchez M, Gérinière L, Perrot E, Souquet PJ, Fontaine-Delaruelle C, David JS, Couraud S. High flow nasal oxygen in frail COVID-19 patients hospitalized in intermediate care units and non-eligible to invasive mechanical ventilation. Respir Med Res. 2023 Nov;84:101026. |
| Dexamethasone treatment | - RECOVERY Collaborative Group; Horby P, Lim WS, Emberson, et al. Dexamethasone in Hospitalized Patients with Covid-19. N Engl J Med. 2021 Feb 25;384(8):693-704 - WHO Rapid Evidence Appraisal for COVID-19 Therapies (REACT) Working Group; Sterne JAC, Murthy S, Diaz JV, et al. Association Between Administration of Systemic Corticosteroids and Mortality Among Critically Ill Patients With COVID-19: A Meta-analysis. JAMA. 2020 Oct 6;324(13):1330-1341. |
| Respiratory rate | - Polydora E, Alexandrou M, Tsipilis S, Athanasiou N, Katsoulis M, Rodopoulou A, et al. Predictors of high flow oxygen therapy failure in COVID-19-related severe hypoxemic respiratory failure. J Thorac Dis. 2022;14(4):851-6. - Raboni SM, Neves VC, Silva RM, Breda GL, Ceregato AC, Broza TP, et al. High-Flow Nasal Cannula Therapy in Patients With COVID-19: Predictive Response Factors. Respir Care. 2022;67(11):1443-51. - Meersseman C, Grolleau E, Freymond N, Wallet F, Gilbert T, Locatelli-Sanchez M, Gérinière L, Perrot E, Souquet PJ, Fontaine-Delaruelle C, David JS, Couraud S. High flow nasal oxygen in frail COVID-19 patients hospitalized in intermediate care units and non-eligible to invasive mechanical ventilation. Respir Med Res. 2023 Nov;84:101026. - Arruda DG, Kieling GA, Melo-Diaz LL. Effectiveness of high-flow nasal cannula therapy on clinical outcomes in adults with COVID-19: A systematic review. Can J Respir Ther. 2023;59:52-65. |
| Oxygen saturation | - Xia J, Zhang Y, Ni L, Chen L, Zhou C, Gao C, et al. High-Flow Nasal Oxygen in Coronavirus Disease 2019 Patients With Acute Hypoxemic Respiratory Failure: A Multicenter, Retrospective Cohort Study. Crit Care Med. 2020;48(11):e1079-e86. - Schmidt F, Nowak L, Obereisenbuchler F, Hetrodt J, Heiss-Neumann M, Schonlebe A, et al. Predicting the effectiveness of high-flow oxygen therapy in COVID-19 patients: a single-centre observational study. Anaesthesiol Intensive Ther. 2022;54(1):12-7. - Meersseman C, Grolleau E, Freymond N, Wallet F, Gilbert T, Locatelli-Sanchez M, Gérinière L, Perrot E, Souquet PJ, Fontaine-Delaruelle C, David JS, Couraud S. High flow nasal oxygen in frail COVID-19 patients hospitalized in intermediate care units and non-eligible to invasive mechanical ventilation. Respir Med Res. 2023 Nov;84:101026. - Arruda DG, Kieling GA, Melo-Diaz LL. Effectiveness of high-flow nasal cannula therapy on clinical outcomes in adults with COVID-19: A systematic review. Can J Respir Ther. 2023;59:52-65. |
| Fraction of inspired oxygen (FiO_2_) | - Mauri T, Carlesso E, Spinelli E, Turrini C, Corte FD, Russo R, et al. Increasing support by nasal high flow acutely modifies the ROX index in hypoxemic patients: A physiologic study. J Crit Care. 2019;53:183-5. - Schmidt F, Nowak L, Obereisenbuchler F, Hetrodt J, Heiss-Neumann M, Schonlebe A, et al. Predicting the effectiveness of high-flow oxygen therapy in COVID-19 patients: a single-centre observational study. Anaesthesiol Intensive Ther. 2022;54(1):12-7. - Meersseman C, Grolleau E, Freymond N, Wallet F, Gilbert T, Locatelli-Sanchez M, Gérinière L, Perrot E, Souquet PJ, Fontaine-Delaruelle C, David JS, Couraud S. High flow nasal oxygen in frail COVID-19 patients hospitalized in intermediate care units and non-eligible to invasive mechanical ventilation. Respir Med Res. 2023 Nov;84:101026. - Arruda DG, Kieling GA, Melo-Diaz LL. Effectiveness of high-flow nasal cannula therapy on clinical outcomes in adults with COVID-19: A systematic review. Can J Respir Ther. 2023;59:52-65. |

# **Table 5. Patient’s characteristics for total cohort and divided between HFNO and COT therapy**

|  | **Total cohort (n=218)** | **HFNO (n=110)** | **COT (n=108)** | **p-value*** | **Missing (n (%))** | |
| --- | --- | --- | --- | --- | --- | --- |
| **Demographics at hospital admission** |  |  |  |  |  | |
| Age (years) | 78 [72-83] | 75 [69-80] | 80 [75-85] | <0.001 | 0 (0) | |
| Sex (male, n (%)) | 135 (62) | 69 (63) | 66 (61) | 0.92 | 0 (0) | |
| Body Mass Index (kg/m^2^) | 27 [24-31] | 28 [24-32] | 26 [24-29] | 0.15 | 15 (7) | |
| Days ill since symptom onset | 6 [4-9] | 7 [5-9] | 5 [4-9] | 0.18 | 6 (3) | |
| 4C mortality score | 13 [11-14] | 13 [11-14] | 12 [11-14] | 0.30 | 47 (22) | |
| Charlson Comorbidity Index (n (%))  0  1  >2 | 19 (9)  75 (34)  124 (57) | 14 (13)  36 (33)  60 (55) | 5 (5)  39 (36)  64 (59) | 0.11 | 0 (0) | |
| Clinical Frailty Scale (n (%))  Fit (1-3)  Vulnerable (4-5)  Frail (6-9) | 47 (22)  101 (46)  70 (32) | 37 (34)  41 (37)  32 (29) | 10 (9)  60 (56)  38 (35) | <0.001 | 0 (0) | |
|  |  |  |  |  |  | |
| **Laboratory at hospital admission** |  |  |  |  |  | |
| CRP (mg/mL) | 94 [48-148] | 90 [49-141] | 95 [46-154] | 0.73 | 0 (0) | |
| Urea (mmol/L) | 9.2 [6.6-12.3] | 8.6 [6.2-11.6] | 9.7 [7.5-12.8] | 0.02 | 7 (3) | |
| Platelet count (10^9^/L) | 175 [129-231] | 177 [130-232] | 169 [131-228] | 0.98 | 2 (0.9) | |
| Lymphocyte count (10^9^/L) | 0.70 [0.54-0.90] | 0.70 [0.50-0.90] | 0.80 [0.60-1.00] | 0.02 | 79 (36) | |
|  |  |  |  |  |  | |
| **Parameters at hospital admission** |  |  |  |  |  | |
| Respiratory rate (breaths/minute) | 24 [20-30] | 25 [20-30] | 24 [20-28] | 0.30 | 1 (0.5) | |
| SpO_2_ (%) | 95 [93-96] | 94 [92-96] | 95 [94-96] | 0.06 | 0 (0) | |
| FiO_2_ categories (n (%)) ‡  1  2  3  4 | 41 (19)  133 (62)  14 (7)  27 (13) | 21 (19)  58 (53)  8 (7)  22 (20) | 20 (19)  75 (71)  6 (6)  5 (5) | 0.004 | 3 (1) | |
| Oxygen device (n (%))  Room air  Nasal cannula 1 L/min  Nasal cannula 2 L/min  Nasal cannula 3 L/min  Nasal cannula 4 L/min  Nasal cannula 5 L/min  Nasal cannula 6 L/min  Air entrainment mask 6 L/min  Air entrainment mask 10 L/min  Non-rebreathing mask 10 L/min  Air entrainment mask 15 L/min  Non-rebreathing mask 15 L/min | 41 (19)  6 (3)  31 (14)  22 (10)  20 (9)  32 (15)  9 (4)  2 (0.9)  11 (5)  3 (1)  11 (5)  27 (12) | 21 (19)  2 (2)  15 (14)  8 (7)  8 (7)  18 (17)  4 (4)  0 (0)  3 (3)  2 (2)  6 (6)  22 (20) | 20 (19)  4 (4)  16 (15)  14 (13)  12 (11)  14 (13)  5 (5)  2 (2)  8 (7)  1 (0.9)  5 (5)  5 (5) | 0.048 | 3 (1) | |
| S/F ratio | 288 [238-359] | 272 [162-352] | 307 [261-363] | 0.03 | 3 (1) | |
| ROX index | 12.2 [8.0-16.5] | 11.5 [6.8-15.9] | 13.2 [9.4-17.2] | 0.03 | 4 (2) | |
|  |  |  |  |  |  | |
| **Treatment during hospital admission** |  |  |  |  |  | |
| Dexamethasone (n (%)) | 190 (87) | 106 (96) | 84 (78) | <0.001 | 0 (0) | |
| Interleukin-6 receptor blockers (n (%)) | 30 (14) | 23 (21) | 7 (7) | 0.004 | 0 (0) | |
|  |  |  |  |  |  | |
| **Prior to HFNO initiation or COT at >6 L/min** |  |  |  |  |  | |
| Hours from hospital admission | 46.1 [3.8-103.1] | 53.3 [7.4-126.2] | 35.3 [1.2-90.8] | 0.02 | 6 (3) | |
| Respiratory rate (breaths/minute) | 28 [22-32] | 28 [23-32] | 28 [22-32] | 0.55 | 0 (0) | |
| SpO_2_ (%) | 93 [91-95] | 92 [89-94] | 94 [93-95] | <0.001 | 0 (0) | |
| FiO_2_ categories (n (%)) ‡  1  2  3  4 | 0 (0)  84 (39)  34 (16)  100 (46) | 0 (0)  18 (16)  20 (18)  72 (66) | 0 (0)  66 (61)  14 (13)  28 (26) | <0.001 | 0 (0) | |
| Oxygen device  Nasal cannula 6 L/min  Air entrainment mask 6 L/min  Air entrainment mask 10 L/min  Non-rebreathing mask 10 L/min  Air entrainment mask 15 L/min  Non-rebreathing mask 15 L/min | 41 (19)  3 (1)  40 (18)  3 (1)  31 (14)  100 (46) | 10 (9)  0 (0)  8 (7)  2 (2)  18 (16)  72 (66) | 31 (29)  3 (3)  32 (30)  1 (0.9)  13 (12)  28 (26) | <0.001 | 0 (0) | |
| S/F ratio | 152 [141-233] | 144 [136-155] | 231 [150-238] | <0.001 | 0 (0) | |
| ROX index | 6.4 [4.8-8.6] | 5.4 [4.4-6.9] | 7.7 [5.4-10.0] | <0.001 | 0 (0) | |
|  |  |  |  |  |  | |
| **Prior to HFNO initiation or COT at maximal level** |  |  |  |  |  | |
| Hours from hospital admission | 60.3 [15.4-124.5] | 53.3 [7.4-126.2] | 64.7 [23.8-122.3] | 0.29 | 6 (3) | |
| Respiratory rate (breaths/minute) | 28 [22-32] | 28 [23-32] | 26 [22-32] | 0.42 | 0 (0) | |
| SpO_2_ (%) | 94 [90-96] | 92 [89-94] | 95 [93-97] | <0.001 | 0 (0) | |
| FiO_2_ categories (n (%)) ‡  1  2  3  4 | 0 (0)  26 (12)  42 (19)  150 (69) | 0 (0)  18 (16)  20 (18)  72 (65) | 0 (0)  8 (7)  22 (20)  78 | 0.13 | 0 (0) | |
| Oxygen device  Nasal cannula 6 L/min  Air entrainment mask 6 L/min  Air entrainment mask 10 L/min  Non-rebreathing mask 10 L/min  Air entrainment mask 15 L/min  Non-rebreathing mask 15 L/min | 10 (5)  0 (0)  16 (7)  2 (0.9)  40 (18)  150 (69) | 10 (9)  0 (0)  8 (7)  2 (2)  18 (16)  72 (65) | 0 (0)  0 (0)  8 (7)  0 (0)  22 (20)  78 (72) | 0.01 | 0 (0) | |
| S/F ratio | 145 [139-154] | 144 [136-155] | 145 [142-152] | 0.13 | 0 (0) | |
| ROX index | 5.5 [4.6-7.0] | 5.4 [4.4-6.9] | 5.6 [4.7-7.1] | 0.50 | 0 (0) | |
|  |  |  |  |  |  | |
| **Outcomes** |  |  |  |  |  | |
| In-hospital mortality (n (%)) | 147 (67) | 70 (64) | 77 (71) | 0.29 | 0 (0) | |
| Cause of death (n (%))  Respiratory failure  Multi-organ failure  Myocardial infarction  Thrombotic event (CVA, PE) | 141 (96)  3 (2)  1 (0.7)  2 (1) | 67 (96)  1 (1)  0 (0)  2 (3) | 74 (96)  2 (3)  1 (1)  0 (0) | 0.31 | 0 (0) | |
| Hospital length of stay [median, IQR]  All  Survivors  Non-survivors | 9 [5-16]  15 [10-21]  6 [4-11] | 11 [6-18]  18 [11-23]  8 [4-14] | 7 [4-12]  13 [8-18]  6 [3-10] | 0.001 | 0 (0) | |
|  |  |  |  |  |  | |
| Data presented as median [interquartile ranges], unless denoted otherwise. *****Using Mann-Whitney U-test for continuous variables and chi-square test for categorical variables ^‡^FiO_2_ divided into three categories: group 1) room air, group 2) nasal oxygen 1-6 L/min or air-entrainment mask 10 L/min, group 3) air-entrainment mask 15 L/min or non-rebreathing mask 10 L/min and group 4) non-rebreathing mask 15 L/min  Abbreviations: COT: Conventional Oxygen Therapy, CVA: Cerebral Vascular Accident, FiO_2_: fraction of inspired oxygen, HFNO: High-Flow Nasal Oxygen, IQR: interquartile range, L: litres, min: minute, n: number, PE: Pulmonary Embolism, ROX index: respiratory oxygenation index (SpO_2_/FiO_2_/respiratory rate), SpO_2_: oxygen saturation, S/F ratio: SpO_2_/FiO_2_ ratio. | | | | | |  |

# **Table 6. Estimates for hospital length of stay**

|  | **Estimates** | **p-value** |
| --- | --- | --- |
| HFNO vs. COT | 0.34 (0.13-0.55) | <0.01 |
| Age (in years) | -0.002 (-0.01-0.01) | 0.74 |
| Clinical Frailty Scale*  Vulnerable vs. Fit  Frail vs. Fit | -0.14 (-0.38-0.09)  -0.09 (-0.34-0.15) | 0.24  0.45 |
| Dexamethasone (yes/no) | 0.24 (-0.03-0.51) | 0.09 |
| SpO_2_ at hospital admission (in %) | 0.01 (-0.02-0.03) | 0.57 |
| FiO_2_ at hospital admission^‡^  Category 2 vs. 1  Category 3 vs. 1  Category 4 vs. 1 | -0.29 (-0.52- -0.06)  -0.20 (-0.61-0.20)  -0.75 (-1.09- -0.42) | 0.01  0.32  <0.001 |
| RR at hospital admission (in breaths/min) | -0.01 (-0.02-0.01) | 0.24 |
|  | | |
| Multivariable linear regression analysis for outcome hospital length of stay  *Clinical Frailty Scale was divided in category 1) fit 1-3, category 2) vulnerable 4-5, category 3) frail 6-9. ^‡^FiO_2_: estimated fraction of inspired oxygen, divided into three categories: group 1) room air, 2) nasal oxygen 1-6 L/min or air-entrainment mask 10 L/min, group 3) air-entrainment mask 15 L/min or non-rebreathing mask 10 L/min and group 4) non-rebreathing mask 15 L/min.  Abbreviations: CI: Confidence Interval, COT: Conventional Oxygen Therapy, HFNO: High-Flow Nasal Oxygen, OR: Odds Ratio, RR: Respiratory rate, SpO_2_: oxygen saturation. | | |

# **Table 7. Patient’s characteristics of total HFNO cohort and divided by survival status**

|  | **Total cohort HFNO** | | **Survivors** | | **Non-survivors** | | **p-value*** | | **Missing (n (%))** | |
| --- | --- | --- | --- | --- | --- | --- | --- | --- | --- | --- |
|  | N = 116 | | N = 42 | | N = 74 | |  | |  | |
| **Demographics at hospital admission** |  | |  | |  | |  | |  | |
| Age (years) | 75 [69-80] | | 74 [66-81] | | 76 [70-79] | | 0.38 | | 0 (0) | |
| Sex (male, n (%)) | 73 (63) | | 29 (69) | | 44 (60) | | 0.41 | | 0 (0) | |
| Body Mass Index (kg/m^2^) | 27 [24-32] | | 28 [24-32] | | 27 [24-32] | | 0.92 | | 6 (5) | |
| Days ill since symptom onset | 7 [4-9] | | 7 [5-12] | | 7 [4-8] | | 0.02 | | 6 (5) | |
| 4C mortality score | 13 [11-14] | | 13 [11-14] | | 12 [11-14] | | 0.47 | | 18 (16) | |
| Charlson Comorbidity Index (n (%))  0  1  >2 | 16 (14)  38 (33)  62 (53) | | 2 (5)  17 (41)  23 (55) | | 14 (19)  21 (28)  39 (53) | | 0.08 | | 0 (0) | |
| Clinical Frailty Scale (n (%))  Fit (1-3)  Vulnerable (4-5)  Frail (6-8) | 39 (35)  41 (37)  32 (29) | | 16 (40)  10 (25)  14 (35) | | 23 (32)  31 (43)  18 (25) | | 0.16 | | 4 (3) | |
|  |  | |  | |  | |  | |  | |
| **Laboratory at hospital admission** |  | |  | |  | |  | |  | |
| CRP (mg/mL) | 90 [49-140] | | 69 [40-139] | | 100 [53-145] | | 0.13 | | 0 (0) | |
| Urea (mmol/L) | 8.6 [6.2-11.7] | | 9.0 [6.4-13.2] | | 8.6 [6.1-11.4] | | 0.64 | | 4 (3) | |
| Platelet count (10^9^/L) | 179 [135-232] | | 183 [150-232] | | 175 [129-230] | | 0.27 | | 0 (0) | |
| Lymphocyte count (10^9^/L) | 0.70 [0.50-0.90] | | 0.70 [0.56-1.00] | | 0.66 [0.50-0.80] | | 0.21 | | 41 (35) | |
|  |  | |  | |  | |  | |  | |
| **Parameters at hospital admission** |  | |  | |  | |  | |  | |
| Respiratory rate (breaths/minute) | 25 [20-30] | | 27 [22-30] | | 24 [20-30] | | 0.31 | | 0 (0) | |
| SpO_2_ (%) | 94 [92-96] | | 95 [92-96] | | 94 [92-96] | | 0.78 | | 0 (0) | |
| FiO_2_ categories (n (%)) ‡  1  2  3  4 | 23 (20)  60 (52)  8 (7)  24 (21) | | 10 (24)  20 (48)  4 (10)  7 (17) | | 13 (18)  40 (54)  4 (5)  17 (23) | | 0.59 | | 1 (0.9) | |
| Oxygen device (n (%))  Room air  Nasal cannula 1 L/min  Nasal cannula 2 L/min  Nasal cannula 3 L/min  Nasal cannula 4 L/min  Nasal cannula 5 L/min  Nasal cannula 6 L/min  Air entrainment mask 6 L/min  Air entrainment mask 10 L/min  Non-rebreathing mask 10 L/min  Air entrainment mask 15 L/min  Non-rebreathing mask 15 L/min | 23 (20)  3 (3)  15 (13)  8 (7)  9 (8)  18 (16)  4 (4)  0 (0)  3 (3)  2 (2)  6 (5)  24 (21) | | 10 (24)  2 (5)  3 (7)  4 (10)  3 (7)  7 (17)  1 (2)  0 (0)  0 (0)  0 (0)  4 (10)  7 (17) | | 13 (18)  1 (1)  12 (16)  4 (5)  6 (8)  11 (15)  3 (4)  0 (0)  3 (4)  2 (3)  2 (3)  17 (23) | | 0.42 | | 1 (0.9) | |
| S/F ratio | 272 [162-354] | | 288 [162-396] | | 268 [167-351] | | 0.50 | | 1 (0.9) | |
| ROX index | 11.6 [6.8-15.9] | | 11.7 [6.7-15.2] | | 11.5 [6.9-17.2] | | 0.97 | | 1 (0.9) | |
|  |  | |  | |  | |  | |  | |
| **Treatment during hospital admission** |  | |  | |  | |  | |  | |
| Dexamethasone (n (%)) | 112 (97) | | 41 (98) | | 71 (96) | | 1.00 | | 0 (0) | |
| Interleukin-6 receptor blockers (n (%)) | 25 (22) | | 11 (26) | | 14 (19) | | 0.50 | | 0 (0) | |
|  |  | |  | |  | |  | |  | |
| **Prior to HFNO initiation** |  | |  | |  | |  | |  | |
| Hours from hospital admission | 51 [6-125] | | 45 [7-126] | | 57 [5-123] | | 0.91 | | 6 (5) | |
| Respiratory rate (breaths/minute) | 28 [23-32] | | 28 [22-31] | | 28 [24-32] | | 0.45 | | 2 (2) | |
| SpO_2_ (%) | 92 [89-94] | | 93 [90-95] | | 92 [88-94] | | 0.03 | | 0 (0) | |
| FiO_2_ categories (n (%)) ‡  1  2  3  4 | 0 (0)  19 (16)  20 (17)  77 (66) | | 0 (0)  10 (24)  11 (26)  21 (50) | | 0 (0)  9 (12)  9 (12)  56 (76) | | 0.02 | | 0 (0) | |
| Oxygen device (n (%))  Nasal cannula 6 L/min  Air entrainment mask 6 L/min  Air entrainment mask 10 L/min  Non-rebreathing mask 10 L/min  Air entrainment mask 15 L/min  Non-rebreathing mask 15 L/min | 10 (9)  0 (0)  9 (8)  2 (2)  18 (16)  77 (66) | | 7 (17)  0 (0)  3 (7)  1 (2)  10 (24)  21 (50) | | 3 (4)  0 (0)  6 (8)  1 (1)  8 (11)  56 (76) | | 0.03 | | 0 (0) | |
| S/F ratio | 144 [136-154] | | 149 [142-181] | | 139 [135-148] | | <0.001 | | 0 (0) | |
| ROX index | 5.4 [4.4-6.9] | | 5.9 [4.9-8.0] | | 5.0 [4.4-6.6] | | 0.03 | | 1 (0.9) | |
|  |  | |  | |  | |  | |  | |
| **Outcomes** |  | |  | |  | |  | |  | |
| Hours on HFNO | 104 [57-187] | | 167 [109-258] | | 73 [42-145] | | <0.001 | | 4 (4) | |
| Hospital length of stay | 11 [6-18] | | 18 [11-23] | | 8 [4-14] | | <0.001 | | 0 (0) | |
|  |  |  | |  | |  | |  | |  |
| Data presented as median [interquartile ranges], unless denoted otherwise. *Using Mann-Whitney U-test for continuous variables and chi-square test for categorical variables ^‡^FiO_2_ divided into three categories: group 1) room air, group 2) nasal oxygen 1-6 L/min or air-entrainment mask 10 L/min, group 3) air-entrainment mask 15 L/min or non-rebreathing mask 10 L/min and group 4) non-rebreathing mask 15 L/min.  Abbreviations: CRP: c-reactive protein, CVA: Cerebral Vascular Accident, FiO_2_: fraction of inspired oxygen, HFNO: High-Flow Nasal Oxygen, L: litres, min: minute, N: number, PE: Pulmonary Embolism, ROX index: respiratory oxygenation index (SpO_2_/FiO_2_/respiratory rate), SpO_2_: oxygen saturation, S/F ratio: SpO_2_/FiO_2_ ratio, T: time point in hours. | | | | | | | | | |  |

# **Table 8. C-statistic of multivariate models, S/F ratio and ROX index for in-hospital mortality at different time points**

**after HFNO start**

|  | C-statistic [95% CI] |  | C-statistic [95% CI] |
| --- | --- | --- | --- |
| Prior to HFNO initiation |  | T = 2h |  |
| Multivariable model* | 0.71 [0.60-0.80] | Multivariable model****** | 0.69 [0.56-0.79] |
| S/F ratio^‡^ | 0.70 [0.59-0.79] | S/F ratio^‡^ | 0.62 [0.51-0.72] |
| ROX index^‡^ | 0.63 [0.52-0.73] | ROX index^‡^ | 0.65 [0.53-0.75] |
|  |  |  |  |
| T = 0h |  | T = 6h |  |
| Multivariable model** | 0.70 [0.60-0.79] | Multivariable model****** | 0.66 [0.55-0.76] |
| S/F ratio^‡^ | 0.64 [0.54-0.74] | S/F ratio^‡^ | 0.61 [0.50-0.71] |
| ROX index^‡^ | 0.63 [0.52-0.73] | ROX index^‡^ | 0.58 [0.47-0.68] |
|  |  |  |  |
| T = 0.5h |  | T = 12h |  |
| Multivariable model** | 0.64 [0.52-0.74] | Multivariable model****** | 0.68 [0.57-0.78] |
| S/F ratio^‡^ | 0.62 [0.50-0.72] | S/F ratio^‡^ | 0.63 [0.52-0.73] |
| ROX index^‡^ | 0.62 [0.50-0.73] | ROX index^‡^ | 0.64 [0.52-0.74] |
|  |  |  |  |
| T = 1h |  | T = 24h |  |
| Multivariable model** | 0.62 [0.50-0.72] | Multivariable model****** | 0.72 [0.61-0.81] |
| S/F ratio^‡^ | 0.61 [0.49-0.71] | S/F ratio^‡^ | 0.68 [0.57-0.77] |
| ROX index^‡^ | 0.60 [0.48-0.71] | ROX index^‡^ | 0.68 [0.57-0.77] |
| *C-statistic for multivariate model based on SpO_2_, respiratory rate and FiO_2_ group divided into three categories: group 1) room air, group 2) nasal oxygen 1-6 L/min or air-entrainment mask 10 L/min, group 3) air-entrainment mask 15 L/min or non-rebreathing mask 10 L/min and group 4) non-rebreathing mask 15 L/min; **C-statistic for multivariate model based on SpO_2_, respiratory rate and FiO_2_; ^‡^C-statistic for univariable model.  Abbreviations: CI: confidence interval, h: hour, HFNO: high-flow nasal oxygen, ROX index: respiratory oxygenation index (SpO_2_/FiO_2_/respiratory rate), S/F ratio: SpO_2_ divided by FiO_2_, T: time point. | | | |

# **References**

1. Charlson ME, Pompei P, Ales KL, MacKenzie CR. A new method of classifying prognostic comorbidity in longitudinal studies: development and validation. J Chronic Dis. 1987;40(5):373-83.

2. Rockwood K, Song X, MacKnight C, Bergman H, Hogan DB, McDowell I, Mitnitski A. A global clinical measure of fitness and frailty in elderly people. CMAJ. 2005;173(5):489-95.
